# Influence of mothers’ nighttime responses on the sleep–wake rhythm of 1-month-old infants

**DOI:** 10.1038/s41598-021-03717-7

**Published:** 2021-12-21

**Authors:** Momoka Kuroda, Yoshiko Suetsugu, Sachiko Iwata, Masahiro Kinoshita, Fumie Fujita, Yoko Sato, Shinji Saitoh, Osuke Iwata, Seiichi Morokuma

**Affiliations:** 1 Department of Health Sciences, Graduate School of Medical Sciences, Kyushu University, Fukuoka, 812-8582 Japan; 2 Department of Pediatrics and Neonatology, Nagoya City University Graduate School of Medical Sciences, Nagoya, Japan; 3 Department of Paediatrics and Child Health, Kurume University School of Medicine, Fukuoka, Japan; 4 School of Nursing, Nishi-Kyushu University, Saga, Japan

**Keywords:** Health care, Health services, Patient education

## Abstract

The purpose of this study was to analyze the influence of the mothers’ nighttime responses on the sleep–wake rhythm of their 1-month-old infants. This study used an anonymous self-administered survey questionnaire with 1133 mothers of 1-month-old infants. The questionnaire investigated basic information about the parents, growth environment of infants, mothers’ sleep patterns during pregnancy, and infants’ sleep patterns at the age of one month. Logistic regression analysis was used to analyze the influence of nighttime responses on the risk of infants sleeping longer during the day than at night. Regarding nighttime response behavior, it was found that immediately picking up 1-month-old infants results in longer sleep during the day than at night (OR 1.616 [1.017 − 2.566], *p* = 0.042), compared to delaying picking up the infant. It was suggested that the stimulation due to picking up an infant may affect sleep–wake rhythm formation.

## Introduction

It has been reported that the regulation of circadian rhythms and formation of the sleep–wake rhythm at one month of age is related to sociality (4 months old), emotional self-regulation (1 year old), attention (4 years old), and anxiety (6 to 13 years old)^1–4^. Thus, the sleep–wake rhythm at one month of age can be considered one of the factors affecting the development of a child.

In contrast, for mothers, unfamiliar child-rearing fatigue is likely to occur one month after giving birth, which makes the development of maternity blues and postpartum depression more likely^5^. Previous studies have reported that sleep time in children and sleep satisfaction in mothers affect postpartum depression^6^. In addition, since the mother’s sleep in the early stages after childbirth is strongly influenced by the sleep schedule of the child^7,8^, early establishment of a sleep–wake rhythm for the child may reduce the risk of mental health problems for the mother. This suggests that it is important to assist in the formation of a healthy sleep–wake rhythm.

Immediately after birth, infants exhibit ultradian rhythms of sleeping and waking that repeat every two to three hours^9^, but due to immature hypothalamic neurodevelopment, which is the center of circadian rhythm and sleep–wake regulation, it is reported that there is no diurnal difference in sleep time^9–11^. Generally, infants acquire a 24-h sleep–wake rhythm one to three months after birth; however, there are considerable individual differences as to when this is established^7,12^.

In children, the sleep–wake rhythm forms under the mutual influence of neural development and environmental factors. Therefore, exposure to environmental factors, such as the light–dark cycle and the mother's lifestyle, may promote or hinder the establishment of the infant's sleep–wake rhythm^12,13^. In addition, it has been reported that the mother’s sleeping habits during pregnancy, child’s time of birth, infant’s age in weeks, mother’s history of childbirth, and breastfeeding influence the formation of the sleep–wake rhythm in infants^8,14^.

In newborns, the phases of sleep are classified into active, quiet, and transitional sleep^15^. Cross-sectional studies indicate that the active / quiet sleep in newborns develops and shifts to REM / non-REM sleep in children and adults^16,17^. Newborns have a larger ratio of active sleep (REM sleep) time than adults. This is because active sleep is important for brain development, and the ratio of active sleep time reduces with brain maturation^18–20^. During active sleep, infants exhibit hand and foot movements, simple vocalizations (groaning, crying), slow twisting of the body, and facial expressions (suckling and frowning), as if they were awake^15^. The mother, misinterpreting it as the baby being awake, may tend to intervene in some way, such as hugging, to put the baby to sleep. Furthermore, as active sleep accounts for much of newborns’ sleep time, many mothers may respond to these active sleep movements. However, intervening with a child’s active sleep in this manner may inhibit the induction of quiet sleep^21^, which may increase daytime sleep and affect the formation and development of the sleep–wake rhythm.

Furthermore, studies that investigated the relationship between nighttime responses to infants and sleep–wake rhythm formation reported that delaying picking up a baby or breastfeeding when they wake up during the night—soon after birth and five weeks after birth—extended nighttime sleep and reduced the frequency of nighttime waking patterns three months after birth^22^. While the previous study investigated three-month-old infants, much is yet to be elucidated about the influence at one month of age, which is before the sleep–wake rhythm is established^22^.

Therefore, we believe that by examining the impact of maternal intervention on the formation of the sleep–wake rhythm of 1-month-old infants during the night and conveying to mothers how to promote sleep–wake rhythm formation in their infants, it would be possible to improve the mental and physical health of both mothers and infants.

## Methods

### Ethics statement

This study was conducted with permission from the Kyushu University and Kurume University Ethics Committee. All experimental protocols were designed according to the Declaration of Helsinki’s ethical principles and performed in accordance with the Ethical Guidelines for Medical and Health Research Involving Human Subjects. The participants provided informed consent after being given explanations about the purpose of the study, the fact that their participation was voluntary, and that withdrawal from the study midway would have no adverse consequences. Using a written document, the participants were informed that their personal information would be handled carefully and used only for the purposes of the study, that they may withdraw their consent at any time before the end of the study, and approximately how much time it would take to complete the experiments and the questionnaires.

### Study population

Five institutions in Fukuoka Prefecture (two tertiary perinatal centers and three private maternity clinics) participated in this study. Mothers who came for a one-month health checkup with their infants between December 2014 and September 2018 were given questionnaires. The infants were healthy, full-term, and one month old. Pre-term and post-term infants were excluded.

The questionnaires asked about basic information (e.g., age of mother and father, mother’s history of childbirth, education history, household income, child’s growth environment (e.g., breastfeeding method, lights-off time, brightness of the room(s) during the day), mother’s sleep schedule during pregnancy (bedtime, period of sleep at night, and regularity of sleep one month before childbirth), and the child’s sleep status at one month after birth (period of daytime sleep and period of nighttime sleep).

Under the growth environment of the child category, the mothers were asked to select from “Breastfeed right away,” “Pick up right away,” “Observe for a while,” and “Other” as responses to the question on “Nighttime intervention for the child.” “Daytime sleep” was the total number of hours slept between 8:00 AM and 8:00 PM, and “Nighttime sleep” was the total number of hours slept between 8:00 PM and 8:00 AM at one month of age. These times were set based on the annual sunrise/sunset times and the general social activity times in the study area in Japan.

### Data and statistical analyses

We excluded cases with missing information about the daytime and nighttime sleep periods of the infant, as this was necessary for analysis. If daytime sleep was longer than nighttime sleep at one month of age, it was assumed that the formation of the sleep–wake rhythm had been delayed^14^. The children who slept more during the day were classified into the “Day sleep” group, while children who slept more during the night or slept equally during the night and day were classified into the “Night sleep” group. The basic characteristics of the “Day sleep” group and the “Night sleep” group were compared using the Mann–Whitney *U* test for quantitative variables and χ2 test for qualitative variables. We then carried out logistic regression analysis to understand the impact of nighttime interventions on the daytime sleep of 1-month-old infants in the “Day sleep” group. As covariates, we used factors considered to impact the formation of a child’s sleep–wake rhythm (mother’s age, mother’s history of childbirth, gestational age, season during which the child was born, breastfeeding method, lights-off time, and mother’s nighttime sleep during pregnancy). The analysis was carried out using the statistical software IBM SPSS version 25, with a significance level of 5%.

## Results

Of the 1,420 cases who responded to the questionnaires, we excluded the responses of 14 cases of premature birth and 273 cases with missing information. Subsequently, data were analyzed for the remaining 1,133 pairs of infants and mothers.

### Study population characteristics

Table 1 shows the basic characteristics of the study population. The mean age of mothers was 32.0 ± 4.8 years and that of fathers was 33.5 ± 5.9 years. Regarding the mothers’ history of childbirth, most of the participants were multipara mothers (n = 619; 54.6%). Majority of participants were college or university graduates (n = 829; 73.2%). In terms of household income, most participants stated that they were “Not comfortable, but satisfied” (n = 841; 74.2%). The family composition was such that most participants were part of a “nuclear family” (n = 926; 81.7%). The mean gestational age of the infants at birth was 39.5 ± 1.1 weeks, and the mean birth weight was 3.078 ± 379 g. The mean age of infants was 33.5 ± 5.9 days.

**Table 1:** Basic characteristics of the study population.

Item	Details	Mean ± SD/number of people (%)
Age of mother (years old)		32.0 ± 4.8
Age of father (years old)		33.5 ± 5.9
History of childbirth	Primipara	472 (41.7)
	Multipara	619 (54.6)
Education history	Middle school	43 (3.8)
	High school	258 (22.8)
	College/University or more	829 (73.2)
Household income	Comfortable	182 (16.1)
	Not comfortable, but satisfied	841 (74.2)
	Not comfortable at all	93 (8.2)
Family composition	Nuclear family	926 (81.7)
	Extended family	147 (13.0)
Gestational week at birth (weeks)		39.5 ± 1.1
Birth weight (g)		3078 ± 379
Age of infant (days)		33.5 ± 5.9

### Sleep status of 1-month-old children

**Figure 1:**
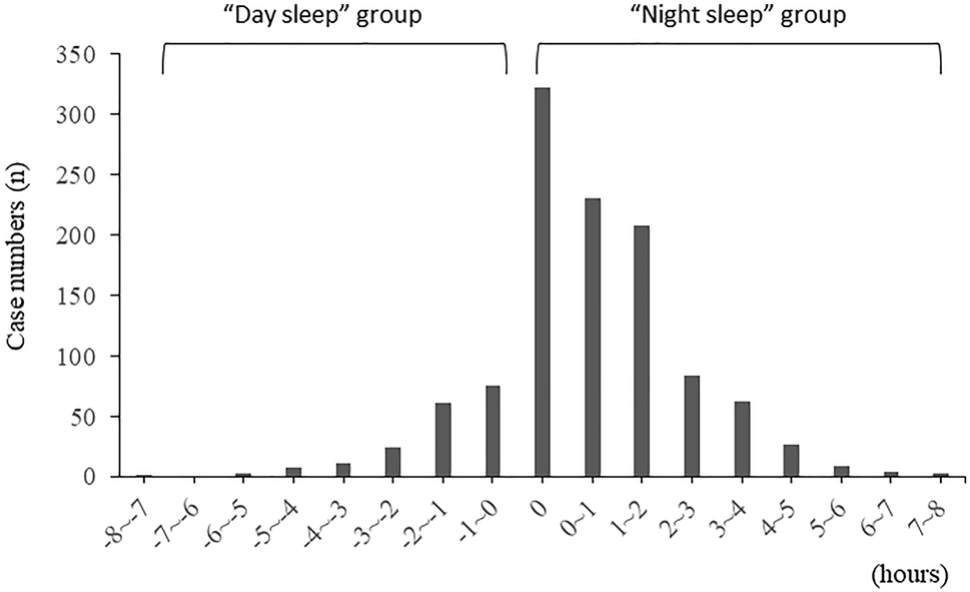
Distribution of difference between daytime sleep and nighttime sleep time. Vertical axis: case numbers, horizontal axis: Nighttime sleep time—Daytime sleep time (h). When Nighttime sleep time minus Daytime sleep time was 0 or more, the infant was classified in the “Night sleep” group.

The mean sleep time of infants was 7.4 ± 1.8 h during the day and 8.3 ± 1.7 h during the night, which meant that nighttime sleep was significantly longer than daytime sleep (*p* < 0.001). There were 183 infants (16.2%) in the “Day sleep” group and 950 infants (83.8%) in the “Night sleep” group, including 322 infants who had equal day sleep time and night sleep time (Fig. 1).

### Comparison between day sleep group and night sleep group

Table 2 shows the characteristics of the “Day sleep” group and the “Night sleep” group. There were significant differences in history of childbirth, season of birth of the infant, feeding method, lights-off time, and nighttime sleep of the mother during pregnancy.

**Table 2:** Characteristics of day sleep group and night sleep group.

Item	Details	n	Mean ± SD/number of people (%)		Type of analysis	*p*
			Day sleep group	Night sleep group		
Age of mother (years old)		1133	32.4 ± 4.8	31.9 ± 4.8	^†^	0.139
Age of father (years old)		1133	33.6 ± 5.0	33.5 ± 5.5	^†^	0.758
History of childbirth	Primipara	1091	88 (50.0)	384 (42.0)	^‡^	0.049*
	Multipara		88 (50.0)	531 (58)		
Mode of delivery	Vaginal delivery	1125	143	800	^‡^	0.104
	Cesarean section		28	99		
	Emergency Cesarean section		11	44		
Education history	Middle school	1130	9 (4.9)	34 (3.6)	^‡^	0.535
	High school		45 (24.6)	213 (22.5)		
	College/University or more		129 (70.5)	700 (73.9)		
Household income	Comfortable	1116	23 (12.8)	159 (17.0)	^‡^	0.346
	Not comfortable, but satisfied		140 (77.8)	701 (74.9)		
	Not comfortable at all		17 (9.4)	76 (8.1)		
Family composition	Nuclear family	1073	156 (90.7)	770 (85.5)	^‡^	0.067
	Extended family		16 (9.3)	131 (14.5)		
Gestational week at birth (weeks)		1133	39.4 ± 1.1	40.0 ± 1.1	^†^	0.365
Birth weight (g)		1133	3082 ± 387	3077 ± 378	^†^	0.86
	< 2500		15	59		
	2500 ≤ , < 4000		165	882	^‡^	0.419
	4000 ≤ (and above)		3	9		
Age of infant (days)		1133	32.9 ± 6.8	33.6 ± 5.8	^†^	0.301
Season of birth of the infant (month) (time of sunrise-sunset in Fukuoka city)	Spring (Mar–May) 6:20 am–6:31 pm	1133	44 (24.0)	**338 (35.6)**	^‡^	0.021*
	Summer (Jun–Aug) 5:08 am–7:32 pm		34 (18.6)	166 (17.5)		
	Fall (Sep–Nov) 6:00 am–6:14 pm		**60 (32.8)**	248 (26.1)		
	Winter (Dec–Feb) 7:18 am–5:15 pm		45 (24.6)	198 (20.8)		
Feeding method	Breastfeeding	1123	82 (45.6)	**509 (54.0)**	^‡^	0.045*
	Breastfeeding and Formula feeding		90 (50.0)	413 (43.8)		
	Formula feeding		8 (4.4)	21 (2.2)		
Lights-off time (baby’s room)	Regularly, by 9:00 pm	1128	42 (23.0)	**376 (39.8)**	^‡^	< 0.001***
	Regularly, after 9:00 pm		**95 (51.9)**	405 (42.9)		
	Irregularly		**46 (25.1)**	164 (17.4)		
Nighttime sleep of the mother during pregnancy		1133	6.3 ± 1.5	6.7 ± 1.4	^†^	0.003**

^†^Mann–Whitney *U*; ^‡^Chi-squared test; *p < 0.05, **p < 0.01, ***p < 0.001

In bold: Adjusted residuals of the chi-square test, frequency is significantly more than the other.

### Impact of nighttime intervention on the sleep of 1-month-old infants

A logistic regression analysis was performed on the impact of mothers’ nighttime responses on the daytime sleep of the infants of the “Day sleep” group (Table 3). Compared to those who responded with, “Observe for a while,” the risk of daytime sleep was significantly higher in those who chose the “Pick up right away” option (OR 1.616 [1.017–2.566]).

**Table 3:** Impact of nighttime responses on the daytime sleep of the infants in the “Day sleep” group.

Nighttime responses for infants	No. of participants	No. of infants of the “day sleep” group		Simple regression model^†^		p	Multivariable model^‡^		p
		n	%	OR	95%CI		OR	95%CI	
Observe for a while	341	53	15.5	Reference			Reference		
Breastfeed right away	440	63	14.3	0.908	[0.611–1.35]	0.63	1.112	[0.712–1.735]	0.64
Pick up right away	239	54	22.6	1.586	[1.041–2.418]	0.03	1.616	[1.017–2.566]	0.04

Logistic regression analysis. Nighttime responses for infants were analyzed by excluding the “Other” response group (n = 113).

*OR* Odds ratio, *CI* Confidence interval.

^†^Only the nighttime response was entered as independent variable.

^‡^Mother’s age, history of childbirth, gestational week at birth, season of birth of the infant, breastfeeding method, lights-off time, and nighttime sleep of the mother during pregnancy were entered as covariates.

## Discussion

We found that the nighttime response of immediately picking up a 1-month-old infant led to increased daytime sleep in those infants.

### Effect of nighttime care on 1-month-old infants’ sleep

When mothers thought their 1-month-old infants had woken up during the night, picking up infants immediately instead of simply observing them was associated with a greater risk of prolonged daytime sleep. We believe this is because the stimulation of being picked up inhibits active sleep among infants.

It is surmised that in most situations, the mother becomes aware that the child has woken up during the night when the infant is crying. However, after birth, mothers are sensitive to the movements of their infants during the night and are likely to wake up in response to these movements, even in the absence of crying^23^. Therefore, mothers can misinterpret such movements as their child having been awakened, even when they are not crying excessively, are not completely awake, or during active sleep. This can be explained by the fact that in the early stages after birth, infants in active sleep tend to exhibit movements that make them appear as if they are awake^15^. When examining the flow from sleep to awakening, not all stimuli during sleep lead to waking up. Depending on the extent of the stimulus, infants either wake up completely or return to sleep^24^. Therefore, a mother may assume that her child has woken up even though the child is still in active sleep. The stimulation from being picked up may cause the child to wake up and, thus, inhibit the child’s active sleep. This may in turn inhibit the induction of quiet sleep^21^ and reduce nighttime sleep. In addition, the active sleep of infants immediately after birth develops the nerves in the suprachiasmatic nucleus and hypothalamus of the brain that control the circadian/sleep–wake rhythm^25^. These studies suggest that inhibition of active sleep may influence the formation of the sleep–wake rhythm. Active sleep in infants, particularly in the early stages after birth, is important for brain development. Therefore, if parents or caregivers believe that their child has woken up during the night, unless there are clear signs of crying, it is better to observe infants for a while before picking them up, rather than picking them up immediately. It is particularly difficult to distinguish between complete awakening and active sleep in the beginning; therefore, using the aforementioned approach can prevent the infant’s active sleep from being inhibited.

James-Roberts et al. studied the link between nighttime responses for infants at birth and five weeks after birth and the quality of sleep in infants at 3 months after birth, and reported that the duration of nighttime sleep at three months of age was longer in infants whose mothers delayed picking up their babies when they had woken up during the night, and indicated that observing and taking time to pick up their child during the night worked positively toward the child’s sleep–wake rhythm^22^. Taking time to respond to an infant at night may also help increase the infant’s self-calming power. It has been reported that the active/quiet sleep cycle in infants is approximately 45 to 60 min/cycle, and physiological signs of awakening manifest between sleep cycles^9^. If the infant is not allowed to sleep after physiological awakening and transition to the next sleep cycle, sleep can be disrupted. In contrast, infants who are allowed the transition naturally to the next sleep cycle learn how to calm themselves and are able to go back to sleep on their own and reinforce their sleep. Considering this, we believe that observing and refraining from immediately picking up the infant when he or she awakes during the night helps improve the infant’s self-calming power and promotes the formation of a proper sleep–wake rhythm.

### Effect of breastfeeding on 1-month-old infants’ sleep

If the mother immediately picks up an awakening child during the night, it can delay the formation of a proper sleep–wake rhythm in the infant; however, there was no relationship between “breastfeeding right away” and the formation of the sleep–wake rhythm. We believe this is because of the difference in the extent of stimulus between picking up and breastfeeding, which leads to the child waking up. It is difficult to define what “awake” is in infants with immature sleep development^24^. Lijowska et al. defined “awake” as a series of events from sighs, startles, and thrashing limb movements, to full arousal^26^. Furthermore, not all stimuli during sleep lead to the infant waking up. Depending on the extent and intensity of the stimulus, infants may either wake up entirely or return to sleep on their own. In this study, we understood that picking up the child, which involves lifting up and swinging the child, is a strong stimulus that leads to complete awakening, in contrast to breastfeeding, which is a low-intensity stimulus that does not lead to complete awakening. The reason we felt that breastfeeding had no impact on the formation of an infant’s sleep–wake rhythm was the possible influence of melatonin in breastmilk. Melatonin is a hypnotic hormone that is produced in the mother's body and transferred to the breastmilk^27,28^. Therefore, we believe that the hypnotic effect of melatonin acts on infants through breastmilk and ensures nighttime sleep time.

### Study strength and limitations

This study derives it strength from its novelty as it is the first to show the influence of mothers’ nighttime responses on the sleep–wake rhythm of 1-month-old infants. The data used in the study were derived from several clinic sites and included primiparas and multiparas while controlling for parity in the analysis. However, this study had a few limitations. This was an observational study, so there could be confounding factors that were not measured and accounted for. Moreover, the participants were evaluated using a self-reported questionnaire, so there could be some bias. Although the number of people who refused to answer the questionnaire was extremely low, the exact number and background of those who refused could not be ascertained. Further, there was insufficient research on the effects of maternal circadian rhythms other than the mothers’ nighttime sleep during pregnancy. Since the questionnaire was answered by the mother, it was assumed that the mother took care of the child at night, but no clear distinction was made as to whether the mother or the father took care of the child. As this study was limited to low-risk mothers and infants with full-term delivery without any obvious complications, we did not examine the impact on high-risk mothers and infants. The investigation was based on responses given to questionnaires; thus, the data we obtained were lacking in detail compared to using sleep charts and actigraphy for gathering sleep data on mothers and infants. While the act of picking up the infant was thought to affect the sleep–wake rhythm by stimulating infant awakening, we cannot confirm the precise nature of the act, since we did not study the process of the infants being picked up. Finally, as this study was conducted in Japan, its generalizability beyond the Japanese context is limited.

## Conclusions

We conducted a questionnaire-based investigation of 1,133 infants and their mothers regarding the impact of mothers’ nighttime responses to their infants on the formation of the sleep–wake rhythm. As a result, we found that the mothers’ act of picking up the infants immediately, when interpreting that the infant had woken up, may affect sleep–wake rhythm formation in infants.
